# Another evidence for a D47N mutation in GJA8 associated with autosomal dominant congenital cataract

**Published:** 2011-09-01

**Authors:** Li Wang, Yi Luo, Wen Wen, Shenghai Zhang, Yi Lu

**Affiliations:** Department of Ophthalmology, Eye and ENT hospital of Fudan University, Shanghai, China

## Abstract

**Purpose:**

To identify the pathogenic gene mutation in a Chinese family with autosomal dominant inherited nuclear cataract.

**Methods:**

After obtained informed consent, detailed ophthalmic examinations were performed, genomic DNAs were obtained from eighteen family members in a four-generation Chinese family with five affected. All exons of candidate genes were amplified by polymerase chain reaction (PCR) and were sequenced performed by bidirectional sequencing. The stability of mutation was predicted with Prediction of Protein Mutant Stability changes (PoPMuSiC). The structure homology modeling of the mutant protein was based on Swiss-Model Serve, and its structure was displayed and compared with human connexin26 using the RasMol software.

**Results:**

By sequencing the encoding regions of the candidate genes, a missence mutation (c.139G>A) was detected in gap junction protein alpha 8 (*GJA8*) gene, which resulted in the substitution of highly conserved aspartic acid by asparagine at codon 47 (p.D47N). The mutation co-segregated with all patients and was absent in 100 normal Chinese controls. PoPMuSiC analysis showed the change in folding free energy upon mutation (ΔΔG) is 0.31 kcal/mol and the mutation p.D47N is destabilizing. The homology modeling showed that the structure of the mutant protein was different with that of human connexin26.

**Conclusions:**

The study identified a missence mutation (c.139G>A) in *GJA8* gene associated with autosomal dominant congenital cataract in a Chinese family. It gave further evidence for *GJA8* associated with congenital cataract.

## Introduction

The lens is an avascular organ, which is composed of a monolayer of cuboidal epithelial cells covering the anterior surface of elongated fibers, which transmits and focuses light images onto the retina. Interior fiber cells, including both primary and secondary fiber cells, undergo a maturation process to eliminate all intracellular organelles, such as the nucleus, mitochondria, endoplasmic reticulum, Golgi apparatus, etc., thereby minimizing light scattering and ensuring lens transparency [1]. The interior mature fibers have an extremely low metabolic activity and depend mainly on the epithelium and peripheral differential fibers for maintenance. Therefore, the lens has developed as a syncytium and a sophisticated cell-cell communication network, which facilitates both an active metabolism and the transport of small metabolites, such as ions, water and secondary messengers [2]. Intercellular gap junction channels provide pathways for metabolic and electrical coupling between cells in the lens. Gap junction channels consist of connexin protein subunits. To date, many connexin (Cx) genes have been found in the mouse genome and the human genome [3]. Mutations in connexin have been identified with various inherited diseases [4], including Cx32 mutation in X-linked Charcot Marie tooth disease, Cx26 and Cx30 mutations in deafness and skin diseases, Cx46 and Cx50 mutations in hereditary cataracts and Cx31 mutation in erythrokeratodermia variabilis (EKV) and hearing impairment with/without peripheral neuropathy.

In our study, we found a missense mutation – the substitution of aspartic acid toasparagine of the codon 47 (p.D47N) in gap junction protein alpha 8 (*GJA8*) associated with autosomal dominant nuclear cataract in a Chinese family.

## Methods

### Clinical evaluation and DNA specimens

**Figure 1:**
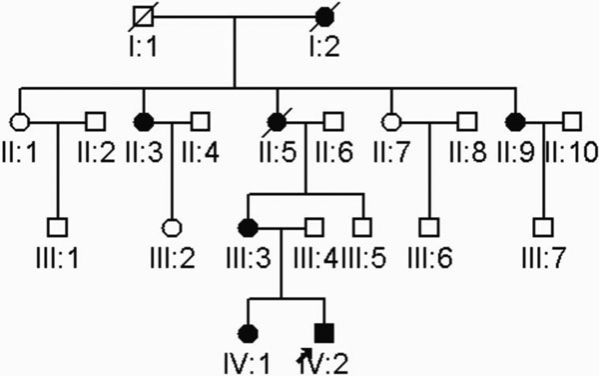
Pedigree of inherited cataract. Squares and circles symbolize males and females, respectively. Clear and blackened symbols denote unaffected and affected individuals, respectively. The arrow indicates the proband.

A four-generation family with autosomal dominant congenital cataract was ascertained (Figure 1). After explanation of the nature and possible consequences of the study, eleven individuals participated in the study. The study was performed with informed consent and following all the guidelines for experimental investigations required by the Institutional Review Board of Eye and EENT Hospital of Fudan University, Shanghai, China. The ophthalmologic examinations, including visual function and dilated slit-lamp examination, were performed by ophthalmologists. Blood samples were collected and leukocyte genomic DNA was extracted.

### Mutation detection

All the exons of candidate genes which associated with autosomal dominant congenital nuclear cataract were amplified by PCR method. The primers used are listed in Appendix 1. The PCR products were sequenced on both directions with an ABI 3130XL Genetic Analyzer (Applied Biosystems, Foster City, CA). The results were analyzed using Chromas (version 2.23) software and compared with the reference sequences in the NCBI gene bank.

### Bioinformatics analysis

The stability of the mutant *GJA8* protein sequences were predicted by Prediction of Protein Mutant Stability changes (PoPMuSiC) [5] with the change in folding free energy upon mutation (ΔΔG). The ΔΔG values are given in kcal/mol. A negative sign corresponds to a mutation predicted as stabilizing. The structure homology modeling of the mutant protein was modeled by Swiss-Model Serve [6], and its structure was displayed and compared with native Cx26 using RasMol software. The structure of native human Cx26 (2zw3) was obtained from the PDB database.

## Results

### Clinical evaluations

**Figure 2:**
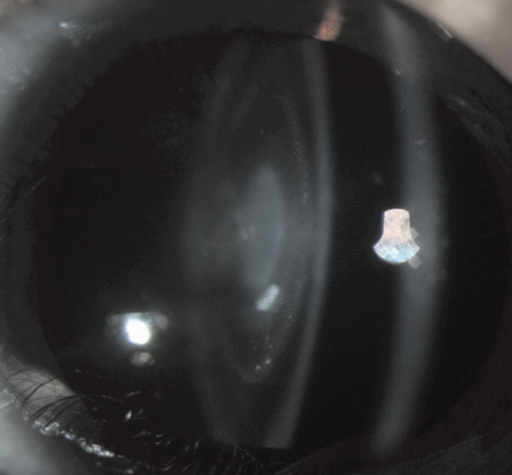
Slit-lamp photograph of the proband. It showed a cataract characterized as a central nuclear opacity of the lens with punctiform opacities.

There were five affected members in this four-generation family (Figure 1). Cataract characterized as bilateral central nuclear cataract with punctiform opacities (Figure 2). There were no other ocular or systemic abnormalities. The affected individuals have had cataract surgery. Autosomal dominant inheritance mode of the cataract was supported by the presence of affected individuals in each of the four generations, and male-to-male transmission.

### Mutation detection

**Figure 3:**
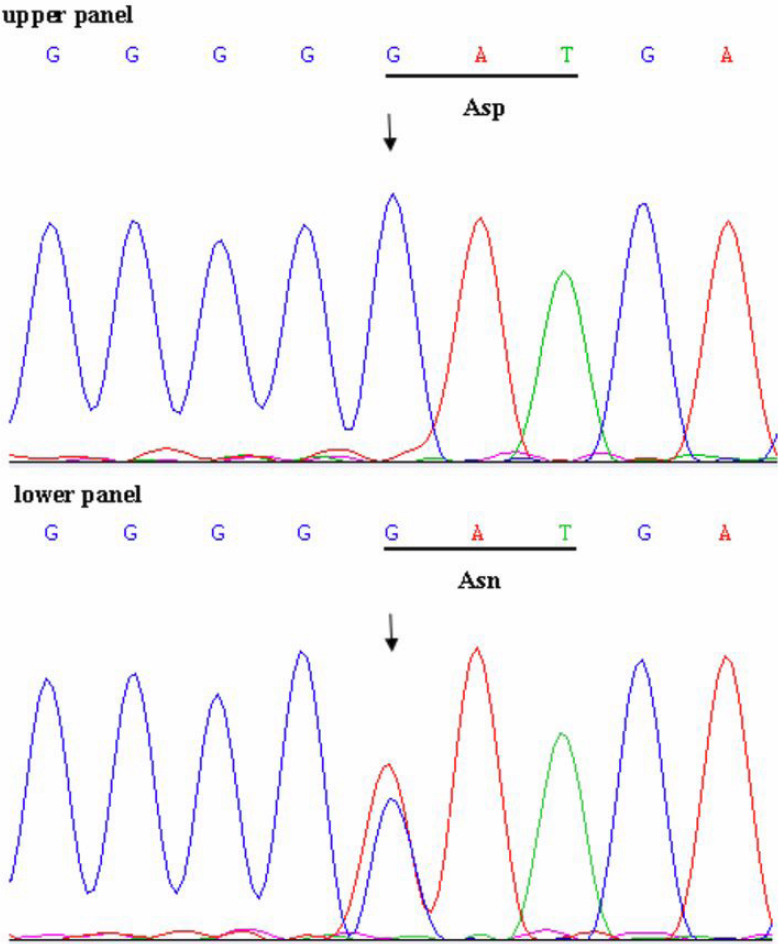
Forward sequence chromatogram of *GJA8*. The arrow indicates the G>A transition. The upper panel is unaffected, the lower panel is affected. The encoded amino acid at codon 47 (underlined) is indicated, GAT encodes Asp (D), and AAT encodes Asn (N).

By bidirectional sequencing of amplified exons of the candidate genes, we found a heterozygous missense mutation, G>A at position 139 in *GJA8* (NM_005267) in affected individuals, but not in unaffected individuals. This change led to the substitution of aspartic acid by asparagine at position 47 (p.D47N; Figure 3). This mutation was not found in 100 unrelated control individuals. No other sequence variant was found.

### Bioinformatics analysis

**Figure 4:**
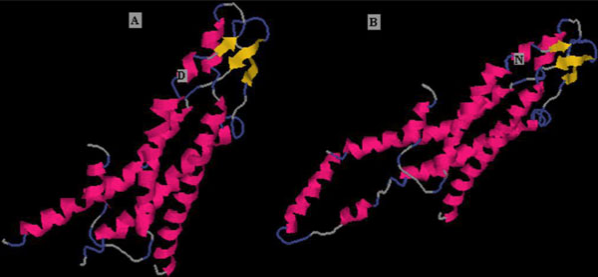
Structure homology modeling and comparison of mutant protein and native human CX26 (2zw3F). **A**: Native human cx26, **B**: Mutant protein CX50. Red, yellow and blue indicate α-helix, β-sheet and β-turn, respectively, white indicates other residues. D and N represent Asp47 and Asn47, respectively.

PoPMuSiC analysis showed that ΔΔG values of the substitution in Cx50 (p.D47N) is 0.31 kcal/mol, which meant that this variant is destabilizing. The homology modeling showed that the second structure of the mutant protein was different with that of human Cx26 (Figure 4).

## Discussion

In a Chinese family with congenital nuclear cataract, we identified a missense mutation c.139G>A in *GJA8*, leading to the substitution of aspartic acid byasparagine (p.D47N). This mutation co-segregated with the phenotype and was not found in 100 unrelated control individuals.

Congenital cataracts are one of the common eye disorders leading to visual impairment or blindness in children worldwide. Congenital cataract may be inherited or familial, either as an isolated form or as a part of a syndrome, such as Nance-Horan syndrome. Along with the development of molecular genetics, more than 20 genes have been identified to be involved in isolated cataract formation, including crystallins, such as αA-crystallin (*CRYAA*), αB-crystallin (*CRYAB*), βA1/A3-crystallin (*CRYBA1/A3*), βA4-crystallin (*CRYBA4*), βB1-crystallin (*CRYBB1*), βB2-crystallin (*CRYBB2*), βB3-crystallin (*CRYBB3*), γC-crystallin (*CRYGC*), γD-crystallin (*CRYGD*), and γS-crystallin (*CRYGS*); membrane transport and channel proteins,such as gap junction protein, alpha 3 (*GJA3*), gap junction protein, alpha 8 (*GJA8*)，intrinsic member protein (*LIM2*), and major intrinsic protein (*MIP*); cytoskeletal proteins, such as beaded filament structural protein 1 (*BFSP1*), beaded filament structural protein 2 (*BFSP2*); transcription factors, such as paired-like homeodomain 3 (*PITX3*), heat shock transcription factor 4 (*HSF4*), Maf-like protein gene (*MAF*), and paired box gene 6 (*PAX6*); others, such as chromatin modifying protein 4B (*CHMP4B*) and Eph-receptor type-A2 (*EPHA2*) [7-12].

Intercellular gap junction channels provide pathways for metabolic and electrical coupling between cells in different tissues. Gap junction channels consist of connexin protein subunits. Connexin proteins have four transmembrane domains with three intracellular regions (the NH_2_-terminus, a cytoplasmic loop and the COOH-terminus) and two extracellular loops (E1 and E2). Six connexin protein subunits oligomerize to form one connexon. A gap junction channel is formed by the docking of extracellular loops of two opposing connexons (hemichannels) in the plasma membrane.

Three isoforms of the connexin gene family are expressed abundantly in the vertebrate lens: *GJA1* (Cx43), *GJA3* (Cx46) and *GJA8* (Cx50) [13-15]. *GJA1* is restrictively expressed in the lens epithelial cells. *GJA3* and *GJA8* are two connexin isoforms in the plasma membrane of fiber cells [16,17]. Many mutations of Cx43 and Cx46 have been reported to be associated with congenital catatact with different phenotype. To date, 19 mutations in the different domain of Cx50 have been identified to contribute to human inherited cataracts (Appendix 2).

Animal models of different connexin knockout and knockin and genetic studies showed *GJA3* and *GJA8* are essential for maintaining lens transparency, and *GJA8* is required for proper fiber cell maturation and control of lens size [18]. Cx46 cannot substitute for Cx50 in lens growth but can prevent lens opacity caused by a lack of Cx50 [19].Electrophysiological studies of intact lenses confirm that Cx46 is essential for the coupling of interior fiber cells while Cx50 is needed for the coupling of both peripheral and interior fiber cells [20-22]. Moreover, Cx50 is clearly necessary for pH-mediated gating of gap junction channels in the differentiating fibers. Mutated Connexins could alter electrical properties of gap junction channels. In No2 mice with dominant cataract, D47A mutant protein of Cx50 was unable to form functional channels and did not inhibit wild type Cx46 or Cx50 junctional conductance in paired *Xenopus* oocytes [23]. A similar point mutation D47N related to human dominant nuclear pulverulent cataracts affect the channel properties in the similiar way [24], D47A and D47N mutants were loss-of-function mutants.

We identified a missense mutation (D47N) in *GJA8* associated with autosomal dominant nuclear cataract in a Chinese family. This finding gives further evidence for *GJA8* in association with congenital cataract. To date, studies of D47N mutant focus on the cellular level, the activity of D47N mutation needs to be further certificated in animal mode.

## Appendix 1.

Primers for PCR amplification of exons of candidate genes and the size of the PCR products. To access the data, click or select the words “Appendix 1.” This will initiate the download of a compressed (pdf) archive that contains the file.

## Appendix 2.

Mutations of GJA8 related with inherited cataract. To access the data, click or select the words “Appendix 2.” This will initiate the download of a compressed (pdf) archive that contains the file.

## References

[1] Bassnett S, Beebe DC (1992). Coincident loss of mitochondria and nuclei during lens fiber cell differentiation.. Dev Dyn.

[2] Mathias RT, Rae JL, Baldo GJ (1997). Physiological properties of the normal lens.. Physiol Rev.

[3] Willecke K, Eiberger J, Degen J, Eckardt D, Romualdi A, Güldenagel M, Deutsch U, Söhl G (2002). Structural and functional diversity of connexin genes in the mouse and human genome.. Biol Chem.

[4] Gerido DA, White TW (2004). Connexin disorders of the ear, skin, and lens.. Biochim Biophys Acta.

[5] Dehouck Y, Grosfils A, Folch B, Gilis D, Bogaerts P, Rooman M (2009). Fast and accurate predictions of protein stability changes upon mutations using statistical potentials and neural networks: PoPMuSiC-2.0.. Bioinformatics.

[6] Arnold K, Bordoli L, Kopp J, Schwede T (2006). The SWISS-MODEL workspace: a web-based environment for protein structure homology modelling.. Bioinformatics.

[7] Reddy MA, Francis PJ, Berry V, Bhattacharya SS, Moore AT (2004). Molecular genetic basis of inherited cataract and associated phenotypes.. Surv Ophthalmol.

[8] Riazuddin SA, Yasmeen A, Yao W, Sergeev YV, Zhang Q, Zulfiqar F, Riaz A, Riazuddin S, Hejtmancik JF (2005). Mutations in betaB3-crystallin associated with autosomal recessive cataract in two Pakistani families.. Invest Ophthalmol Vis Sci.

[9] Sun H, Ma Z, Li Y, Liu B, Li Z, Ding X, Gao Y, Ma W, Tang X, Li X, Shen Y (2005). Gamma-S crystallin gene (CRYGS) mutation causes dominant progressive cortical cataract in humans.. J Med Genet.

[10] Ramachandran RD, Perumalsamy V, Hejtmancik JF (2007). Autosomal recessive juvenile onset cataract associated with mutation in BFSP1.. Hum Genet.

[11] Shiels A, Bennett TM, Knopf HL, Yamada K, Yoshiura K, Niikawa N, Shim S, Hanson PI (2007). CHMP4B, a novel gene for autosomal dominant cataracts linked to chromosome 20q.. Am J Hum Genet.

[12] Shiels A, Bennett TM, Knopf HL, Maraini G, Li A, Jiao X, Hejtmancik JF (2008). The EPHA2 gene is associated with cataracts linked to chromosome 1p.. Mol Vis.

[13] Beyer EC, Paul DL, Goodenough DA (1987). Connexin43: a protein from rat heart homologous to a gap junction protein from liver.. J Cell Biol.

[14] Paul DL, Ebihara L, Takemoto LJ, Swenson KI, Goodenough DA (1991). Connexin46, a novel lens gap junction protein, induces voltage-gated currents in nonjunctional plasma membrane of Xenopus oocytes.. J Cell Biol.

[15] White TW, Bruzzone R, Goodenough DA, Paul DL (1992). Mouse Cx50, a functional member of the connexin family of gap junction proteins, is the lens fiber protein MP70.. Mol Biol Cell.

[16] Gong X, Li E, Klier G, Huang Q, Wu Y, Lei H, Kumar NM, Horwitz J, Gilula NB (1997). Disruption of alpha3 connexin gene leads to proteolysis and cataractogenesis in mice.. Cell.

[17] Rong P, Wang X, Niesman I, Wu Y, Benedetti LE, Dunia I, Levy E, Gong X (2002). Disruption of Gja8 (alpha8 connexin) in mice leads to microphthalmia associated with retardation of lens growth and lens fiber maturation.. Development.

[18] Gong X, Cheng C, Xia CH (2007). Connexins in lens development and cataractogenesis.. J Membr Biol.

[19] White TW (2002). Unique and redundant connexin contributions to lens development.. Science.

[20] Baldo GJ, Gong X, Martinez-Wittinghan FJ, Kumar NM, Gilula NB, Mathias RT (2001). Gap junctional coupling in lenses from alpha(8) connexin knockout mice.. J Gen Physiol.

[21] Gong X, Baldo GJ, Kumar NM, Gilula NB, Mathias RT (1998). Gap junctional coupling in lenses lacking alpha3 connexin.. Proc Natl Acad Sci USA.

[22] Martinez-Wittinghan FJ, Sellitto C, White TW, Mathias RT, Paul D, Goodenough DA (2004). Lens gap junctional coupling is modulated by connexin identity and the locus of gene expression.. Invest Ophthalmol Vis Sci.

[23] Xu X, Ebihara L (1999). Characterization of a mouse Cx50 mutation associated with the No2 mouse cataract.. Invest Ophthalmol Vis Sci.

[24] Arora A, Minogue PJ, Liu X, Addison PK, Russel-Eggitt I, Webster AR, Hunt DM, Ebihara L, Beyer EC, Berthoud VM, Moore AT (2008). A novel connexin50 mutation associated with congenital nuclear pulverulent cataracts.. J Med Genet.

[25] Shiels A, Mackay D, Ionides A, Berry V, Moore A, Bhattacharya S (1998). A missense mutation in the human connexin50 gene (GJA8) underlies autosomal dominant “zonular pulverulent” cataract, on chromosome 1q.. Am J Hum Genet.

[26] Pal JD, Berthoud VM, Beyer EC, Mackay D, Shiels A, Ebihara L (1999). Molecular mechanism underlying a Cx50-linked congenital cataract.. Am J Physiol.

[27] Berry V, Mackay D, Khaliq S, Francis PJ, Hameed A, Anwar K, Mehdi SQ, Newbold RJ, Ionides A, Shiels A, Moore T, Bhattacharya SS (1999). Connexin 50 mutation in a family with congenital “zonular nuclear” pulverulent cataract of Pakistani origin.. Hum Genet.

[28] Polyakov AV, Shagina IA, Khlebnikova OV, Evgrafov OV (2001). Mutation in the connexin 50 gene (GJA8) in a Russian family with zonular pulverulent cataract.. Clin Genet.

[29] Graw J, Schmidt W, Minogue PJ, Rodriguez J, Tong JJ, Klopp N, Illig T, Ebihara L, Berthoud VM, Beyer EC (2009). The GJA8 allele encoding CX50I247M is a rare polymorphism, not a cataract-causing mutation.. Mol Vis.

[30] Willoughby CE, Arab S, Gandhi R, Zeinali S, Arab S, Luk D, Billingsley G, Munier FL, Héon E (2003). A novel GJA8 mutation in an Iranian family with progressive autosomal dominant congenital nuclear cataract.. J Med Genet.

[31] Ma ZW, Zheng JQ, Li J, Li XR, Tang X, Yuan XY, Zhang XM, Sun HM (2005). Two novel mutations of connexin genes in Chinese families with autosomal dominant congenital nuclear cataract.. Br J Ophthalmol.

[32] Arora A, Minogue PJ, Liu X, Reddy MA, Ainsworth JR, Bhattacharya SS, Webster AR, Hunt DM, Ebihara L, Moore AT, Beyer EC, Berthoud VM (2006). A novel GJA8 mutation is associated with autosomal dominant lamellar pulverulent cataract: further evidence for gap junction dysfunction in human cataract.. J Med Genet.

[33] Vanita V, Singh JR, Singh D, Varon R, Sperling K (2008). A mutation in GJA8 (p.P88Q) is associated with “balloon-like” cataract with Y-sutural opacities in a family of Indian origin.. Mol Vis.

[34] Devi RR, Vijayalakshmi P (2006). Novel mutations in GJA8 associated with autosomal dominant congenital cataract and microcornea.. Mol Vis.

[35] Vanita V, Hennies HC, Singh D, Nürnberg P, Sperling K, Singh JR (2006). A novel mutation in GJA8 associated with autosomal dominant congenital cataract in a family of Indian origin.. Mol Vis.

[36] Ponnam SP, Ramesha K, Tejwani S, Ramamurthy B, Kannabiran C (2007). Mutation of the gap junction protein alpha 8 (GJA8) gene causes autosomal recessive cataract.. J Med Genet.

[37] Hansen L, Yao W, Eiberg H, Kjaer KW, Baggesen K, Hejtmancik JF, Rosenberg T (2007). Genetic heterogeneity in microcornea-cataract: five novel mutations in CRYAA, CRYGD, and GJA8.. Invest Ophthalmol Vis Sci.

[38] Schmidt W, Klopp N, Illig T, Graw J (2008). A novel GJA8 mutation causing a recessive triangular cataract.. Mol Vis.

[39] Lin Y, Liu NN, Lei CT, Fan YC, Liu XQ, Yang Y, Wang JF, Liu B, Yang ZL (2008). A novel GJA8 mutation in a Chinese family with autosomal dominant congenital cataract.. Zhonghua Yi Xue Yi Chuan Xue Za Zhi..

[40] Yan M, Xiong C, Ye SQ, Chen Y, Ke M, Zheng F, Zhou X (2008). A novel connexin 50 (GJA8) mutation in a Chinese family with a dominant congenital pulverulent nuclear cataract.. Mol Vis.

[41] Vanita V, Singh JR, Singh D, Varon R, Sperling K (2008). A novel mutation in GJA8 associated with jellyfish-like cataract in a family of Indian origin.. Mol Vis.

[42] Wang K, Wang B, Wang J, Zhou S, Yun B, Suo P, Cheng J, Ma X, Zhu S (2009). A novel GJA8 mutation (p.I31T) causing autosomal dominant congenital cataract in a Chinese family.. Mol Vis.

[43] Hu S, Wang B, Zhou Z, Zhou G, Wang J, Ma X, Qi Y (2010). A novel mutation in GJA8 causing congenital cataract-microcornea syndrome in a Chinese pedigree.. Mol Vis.

[44] Gao X, Cheng J, Lu C, Li X, Li F, Liu C, Zhang M, Zhu S, Ma X (2010). A novel mutation in the connexin 50 gene (GJA8) associated with autosomal dominant congenital nuclear cataract in a Chinese family.. Curr Eye Res.

